# Complete chloroplast genome of *Daphne pseudomezereum* var. *koreana* (Thymelaeaceae)

**DOI:** 10.1080/23802359.2023.2179356

**Published:** 2023-02-25

**Authors:** Su-Chang Yoo, Jongsun Park, Sang-Hun Oh

**Affiliations:** a Department of Biology, Daejeon University, Daejeon, Republic of Korea; b InfoBoss Inc, Seoul, Republic of Korea; c InfoBoss Research Center, Seoul, Republic of Korea

**Keywords:** Chloroplast genome, *Daphne pseudomezereum* var. *koreana*, Korea, Thymelaeaceae

## Abstract

*Daphne pseudomezereum* A. Gray var. *koreana* (Nakai) Hamaya is a shrub distributed in high mountains in Japan and Korea and is used as a medicinal plant. The complete chloroplast genome of *D. pseudomezereum* var. *koreana* is 171,152 bp long with four subregions consisting of a large single-copy region (84,963 bp), a small single-copy region (41,725 bp), and a pair of inverted repeats (2739 bp). The genome includes 139 genes (93 protein-coding genes, eight rRNAs, and 38 tRNAs). Phylogenetic analyses show that *D. pseudomezereum* var. *koreana* is nested within the *Daphne* clade in the narrow sense and that it forms a distinct lineage.

## Introduction

*Daphne pseudomezereum* A. Gray var. *koreana* (Nakai) Hamaya (Bull. Tokyo Univ. Forest. 55: 72, 1959) is a shrub distributed in high mountains in Japan and Korea (Oh and Hong 2015). As a member of Thymelaeaceae, which includes many ecologically important species for ornamentals, timber, papermaking, and medicine (Herber 2003), *D. pseudomezereum* can also be used as a medicinal plant for chronic skin diseases and rheumatism (Konishi et al. 1993). Plants of *D. pseudomezereum* var. *koreana* have rarely been collected, as they are uncommon. For this reason, little is known about the species. Detailed and accurate information on its phylogenetic position and better knowledge of the genome of the species will be useful for understanding its genetic diversity and for evaluating its phytochemical composition for medicinal purposes.

## Materials and methods

**Figure 1.**
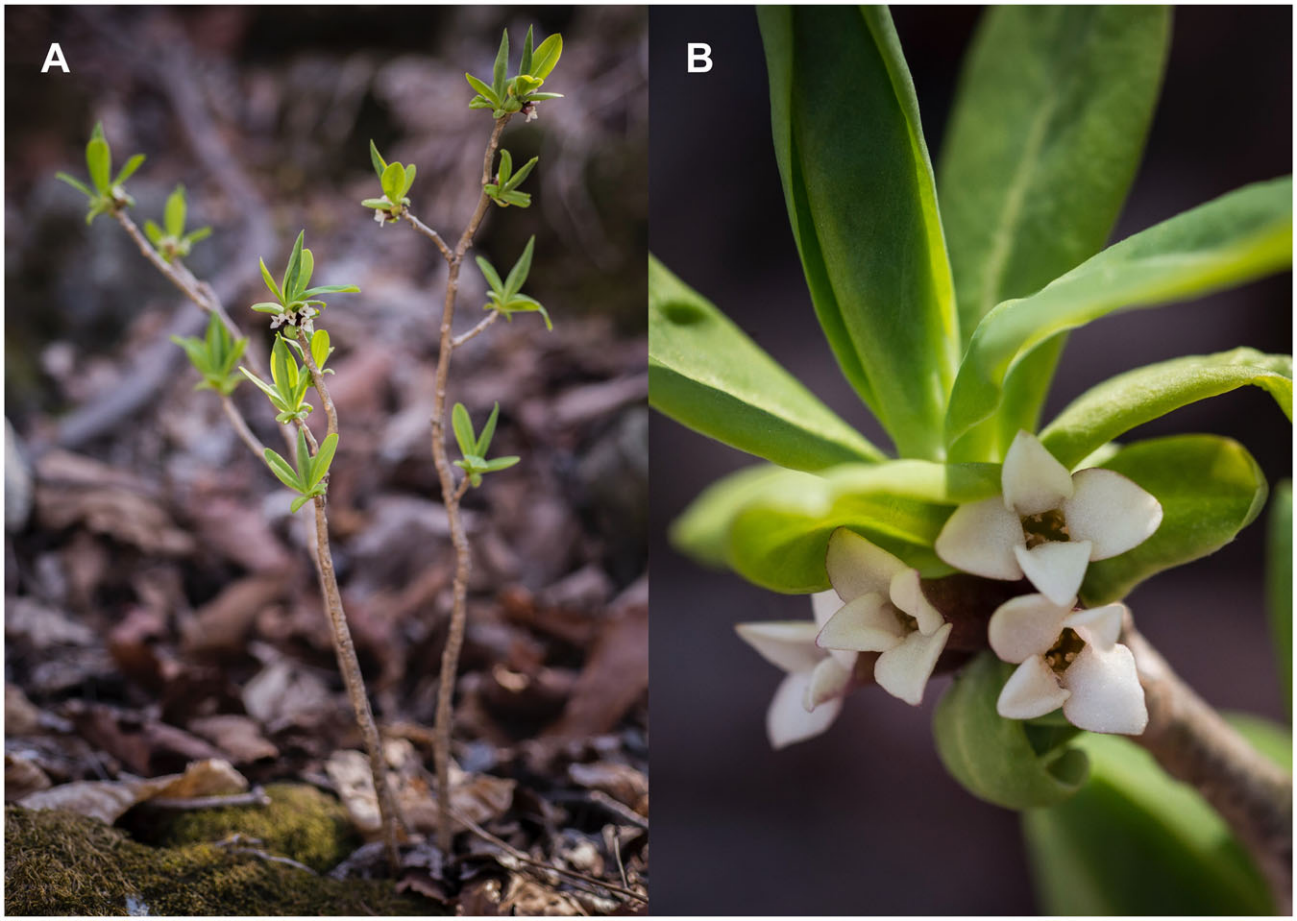
Photograph of *Daphne pseudomezereum* var. *koreana*. (A) Habit. (B) Flowers. Photo credit: Jae-Jin Lee.

**Figure 2.**
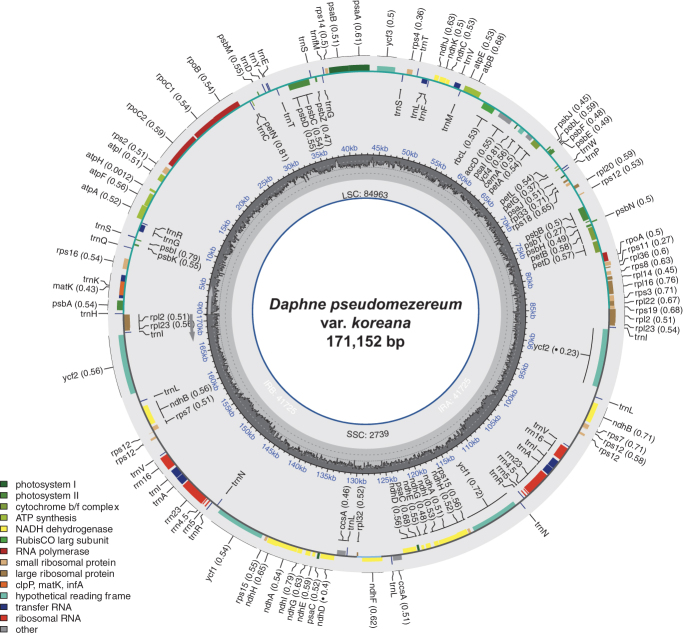
Circular map of the complete chloroplast genome of *Daphne pseudomezereum* var. *koreana*. The center of the map indicates the name of the species and the length of its chloroplast genome. Going outward, the first circle shows LSC, SSC, IRa, and IRb with their length. The second circle displays the GC ratio depicted as the proportion of the shaded parts of each section. The third circle shows the gene names with the colors based on their functional categories provided in the lower left of the circular map. Genes inside the circle are transcribed in a clockwise direction, and those outside are in a counterclockwise direction.

The sample of *D. pseudomezereum* var. *koreana* (Figure 1) were collected in Sangye-ri, Okgye-myeon, Gangneung-si, Gangwon-do, Republic of Korea (37°32′48″N, 128°51'31″E). A voucher specimen was deposited in the Daejeon University Herbarium (TUT: https://www.dju.ac.kr/biosci/depart/profileView.do?mi=2253, contact person: Sang-Hun Oh, soh42@dju.kr) under the voucher number *Lee 7891*. Total DNA was isolated from fresh leaves of the species using a DNeasy Plant Mini Kit (QIAGEN, Hilden, Germany). A small portion (3 ul) of the extracted DNA was run in 1% agarose gel to examine the quality. The concentration of DNA was measured with an Invitrogen Qubit fluorometer. An amount of 1 microgram of DNA was used to reconstruct the sequencing library. We used an Illumina TruSeq Nano DNA Library Preparation Kit (Illumina, San Diego, CA) following the manufacturer’s recommendations. The sequencing library was analyzed using NovaSeq6000 at Macrogen Inc., Korea. The resulting 7.34-Gbp raw sequences were filtered using the Trimmomatic tool (v0.33) (Bolger et al. 2014). The chloroplast genome was *de novo* assembled with Velvet v1.2.10 (Zerbino and Birney 2008) and gaps were closed using GapCloser v1.12 (Zhao et al. 2011). The genome sequence was confirmed by aligning all raw reads against the assembled genome using BWA v0.7.17 and SAMtools v1.9 (Li et al. 2009; Li 2013) in the environment of Genome Information System (GeIS; https://geis.infoboss.co.kr/). Geneious Prime^®^ v2020.2.4 (Biomatters Ltd., Auckland, New Zealand) was used for annotation based on the *Daphne genkwa* chloroplast (MT754180) (Yoo et al. 2021) and read coverage depth map (Supplementary Figure 1). A circular map of the chloroplast genome (Figure 2) and a schematic map of the cis- and trans-splicing genes (Supplementary Figures 2 and 3) were generated by CPGView (Liu et al. 2023).

**Figure 3.**
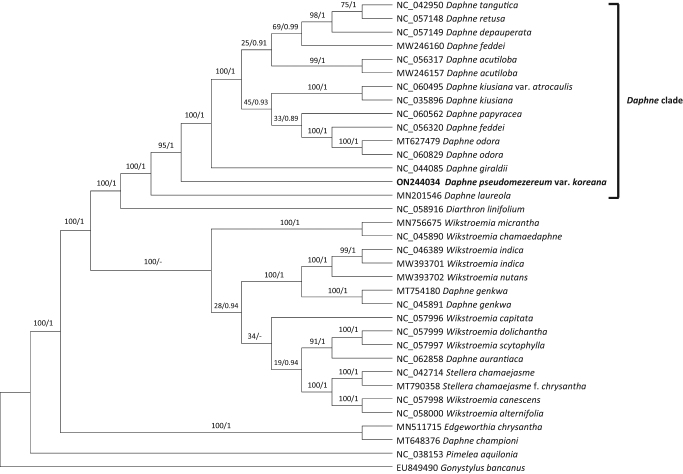
Phylogenetic analyses of 35 whole chloroplast genomes of *Daphne* and closely related species with *Pimelea* and *Gonystylus* as outgroups using the maximum likelihood (ML) and Bayesian inference (BI) methods. The following sequences are used: EU849490 (unpublished), MN201546 (Könyves et al. 2019), MN511715 (Qian et al. 2021), MN756675 (He et al. 2021), MT627479 (Lee et al. 2022), MT648376 (Lee et al. 2022), MT754180 (Yoo et al. 2021), MT790358 (Liang et al. 2020), MW246157 (unpublished), MW246160 (unpublished), MW393701 (unpublished), MW393702 (unpublished), NC_035896 (Cho et al. 2018), NC_038153 (Foster et al. 2018), NC_042714 (Yun et al. 2019), NC_042950 (Yan et al. 2019), NC_044085 (Yan et al. 2019), NC_045890 (Qian et al. 2020), NC_045891 (unpublished), NC_046389 (Qian and Zhang 2019), NC_056317 (unpublished), NC_056320 (unpublished), NC_057148 (Yan et al. 2021), NC_057149 (unpublished), NC_057996 (He et al. 2021), NC_057997 (He et al. 2021), NC_057998 (He et al. 2021), NC_057999 (He et al. 2021), NC_058000 (He et al. 2021), NC_058916 (Kim et al. 2021), NC_060495 (Lee et al. 2022), NC_060562 (Lee et al. 2022), NC_060829 (unpublished), NC_062858 (unpublished), ON244034 (this study). The phylogenetic tree was drawn based on the ML tree. The numbers above the branches indicate bootstrap support values of ML and the posterior probability from BI.

In total, 35 whole chloroplast genomes of *Daphne* and its closely related species (Herber 2003) were included in phylogenetic analyses using the maximum likelihood (ML) and Bayesian inference (BI) methods to infer the phylogenetic position of the chloroplast genome of *D. pseudomezereum* var. *koreana. Pimelea* and *Gonystylus* were used as outgroups. A heuristic search was conducted with nearest-neighbor interchange branch swapping, the Tamura-Nei model, and uniform rates among sites to construct the ML phylogenetic tree with default values for other options using MEGA X (Kumar et al. 2018). A bootstrap analysis with 1000 pseudoreplicates was also conducted with the same search options. The BI tree was constructed using MrBayes v3.2.6 (Ronquist et al. 2012). The GTR model with gamma rates was used as a molecular model. A Markov-chain Monte Carlo algorithm was employed for 1,000,000 generations, sampling trees every 200 generations, with four chains running simultaneously.

## Results

The chloroplast genome of *D. pseudomezereum* var. *koreana* (GenBank accession: ON244034) is 171,152 bp long (Figure 2). The overall GC ratio is 36.5%. The genome has four subregions consisting of 84,963 bp of a large single-copy region (LSC; 49.6%) and 2739 bp of a small single-copy (SSC; 1.6%) region separated by 41,725 bp of each of two inverted repeat regions (IRs; 48.8%). It contains 139 genes (93 protein-coding genes, eight rRNAs, and 38 tRNAs); 28 genes (16 protein-coding gene, four rRNAs, and eight tRNAs) are duplicated in IR regions (Figure 2).

The phylogenetic tree showed that *D. pseudomezereum* var. *koreana* is nested within the *Daphne* clade, forming a distinct lineage within the clade (Figure 3). It was sister to a large subclade that includes most species of *Daphne*, such as *D. giraldii* and *D. tangutica* (Figure 3).

## Discussion and conclusion

The chloroplast genome of *D. pseudomezereum* var. *koreana* exhibits long IR regions and a short SSC region compared to a typical chloroplast genome of angiosperms (Palmer et al. 1988; Cosner et al. 2004). The expansion of IR associated with the shortening of SSC is also found in other species of Thymelaeaceae, such as *Daphne kiusiana* (Cho et al. 2018) and *Daphne laureola* (Könyves et al. 2019) as well as *Aquilaria sinensis* (Wang et al. 2016), *Stellera chamaejasme* (Yun et al. 2019), and *Wikstroemia chamaedaphne* (Qian et al. 2020). It appears that the events of IR expansion and contraction should have occurred multiple times within Thymelaeaceae, and more chloroplast genomes for various taxa are needed to understand the pattern of chloroplast evolution in the family.

The phylogenetic tree indicates that the position of newly determined chloroplast genome of *D. pseudomezereum* var. *koreana* is consistent with morphology (Oh and Hong 2015; Lee J-J and Oh 2017)), placed within the core *Daphne* clade. The phylogenetic analysis suggests that the *Daphne* should be narrowly redefined to indicate the core *Daphne* clade (Figure 3), as the traditional circumscription of the genus *Daphne* (Herber 2003; Wang et al. 2007; Oh and Hong 2015) is polyphyletic. It also suggests that *Wikstroemia* should expand to include *D. genkwa*, *D. aurantiaca*, and *Stellera chamaejasme* and that *D. championi* is sister to *Edgeworthia chrysantha* (Figure 3). Our results, consistent with previous analyses (Yoo et al. 2021; Lee SY et al. 2022), indicate that further detailed systematic studies of *Daphne* and its closely related groups with more taxon sampling are needed. This report contributes to the understanding of the chloroplast genetic information of *D. pseudomezereum* var. *koreana* to provide additional data for future research to reconstruct the evolutionary relationships and for the establishment of a sound classification system of *Daphne* and its closely related groups and ultimately to develop molecular markers for the medicinal plant.

## Supplementary Material

Supplemental Material

## Data Availability

The chloroplast genome sequence can be accessed *via* accession number ON244034 in GenBank of NCBI at https://www.ncbi.nlm.nih.gov. The associated BioProject, SRA, and Bio-Sample numbers are PRJNA835716, SAMN28108557, and SRR19117239, respectively.
