# Supplementary figures and images for: Complete chloroplast genome of Daphne pseudomezereum var. koreana (Thymelaeaceae)
Source: Mitochondrial DNA B Resour. 2023 Feb 25;8(2):305–9. doi: 10.1080/23802359.2023.2179356 (PMC9970196; doi:10.1080/23802359.2023.2179356)

## Slide 1
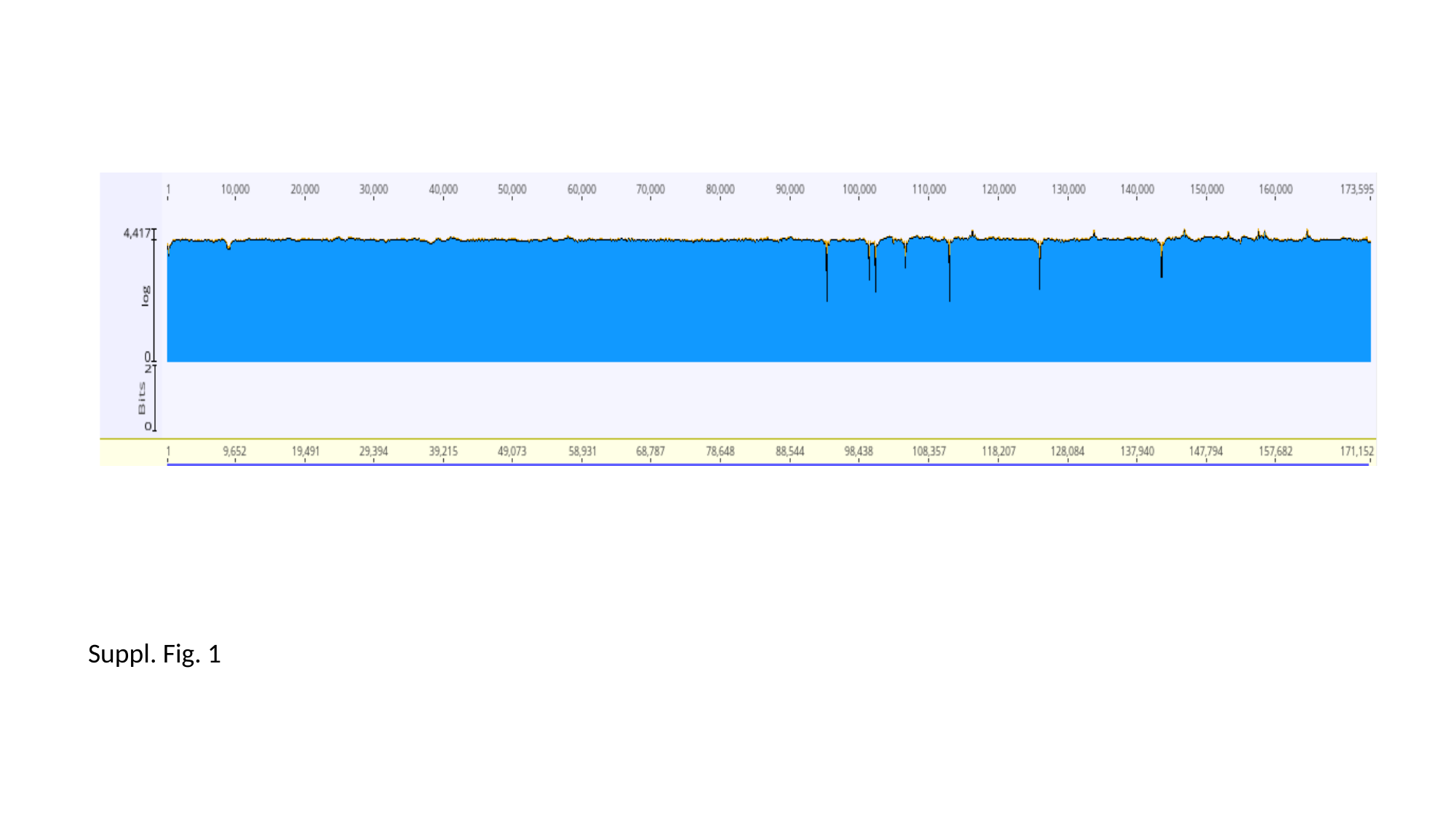

Suppl. Fig. 1

## Slide 2
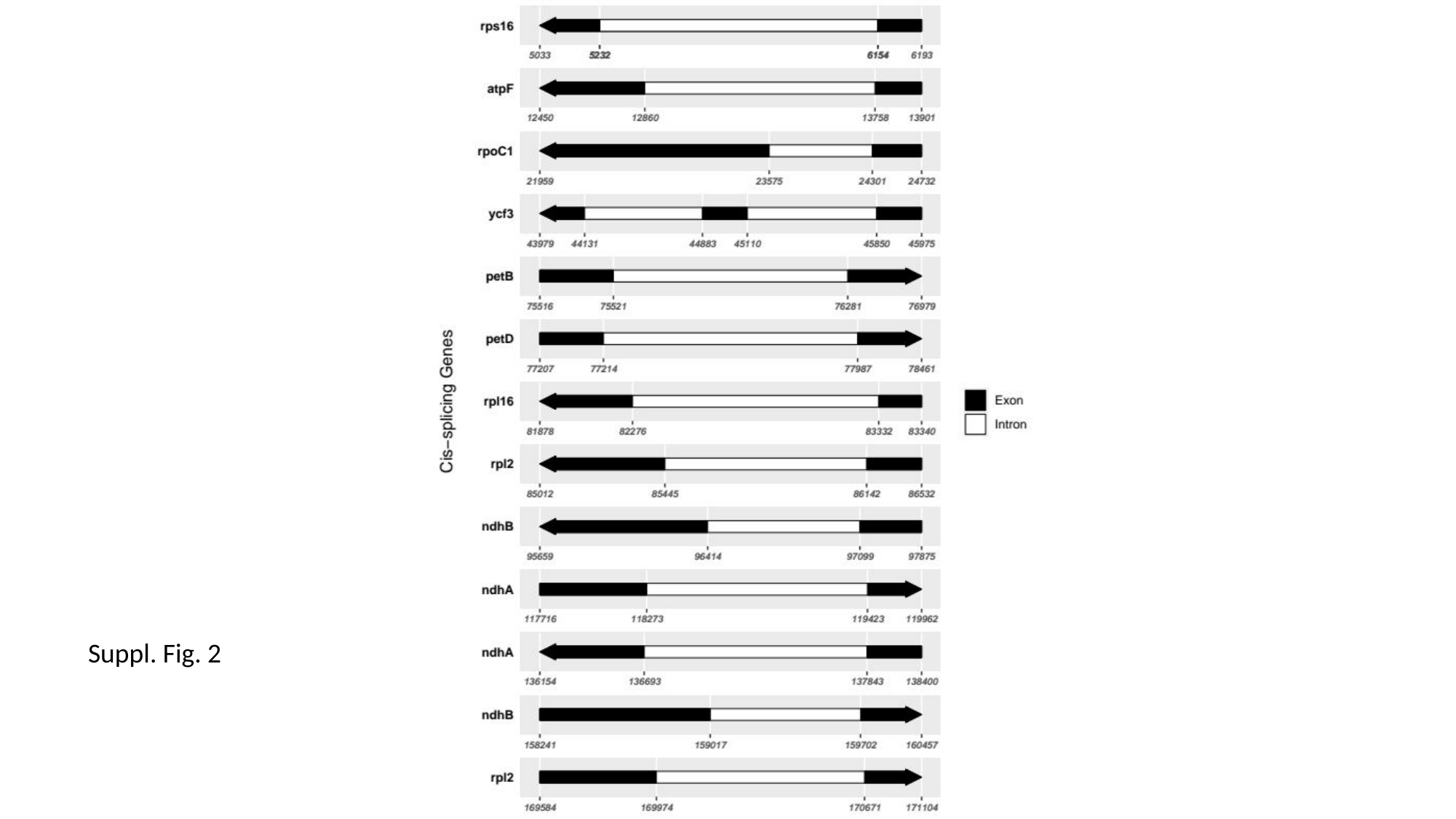

Suppl. Fig. 2

## Slide 3
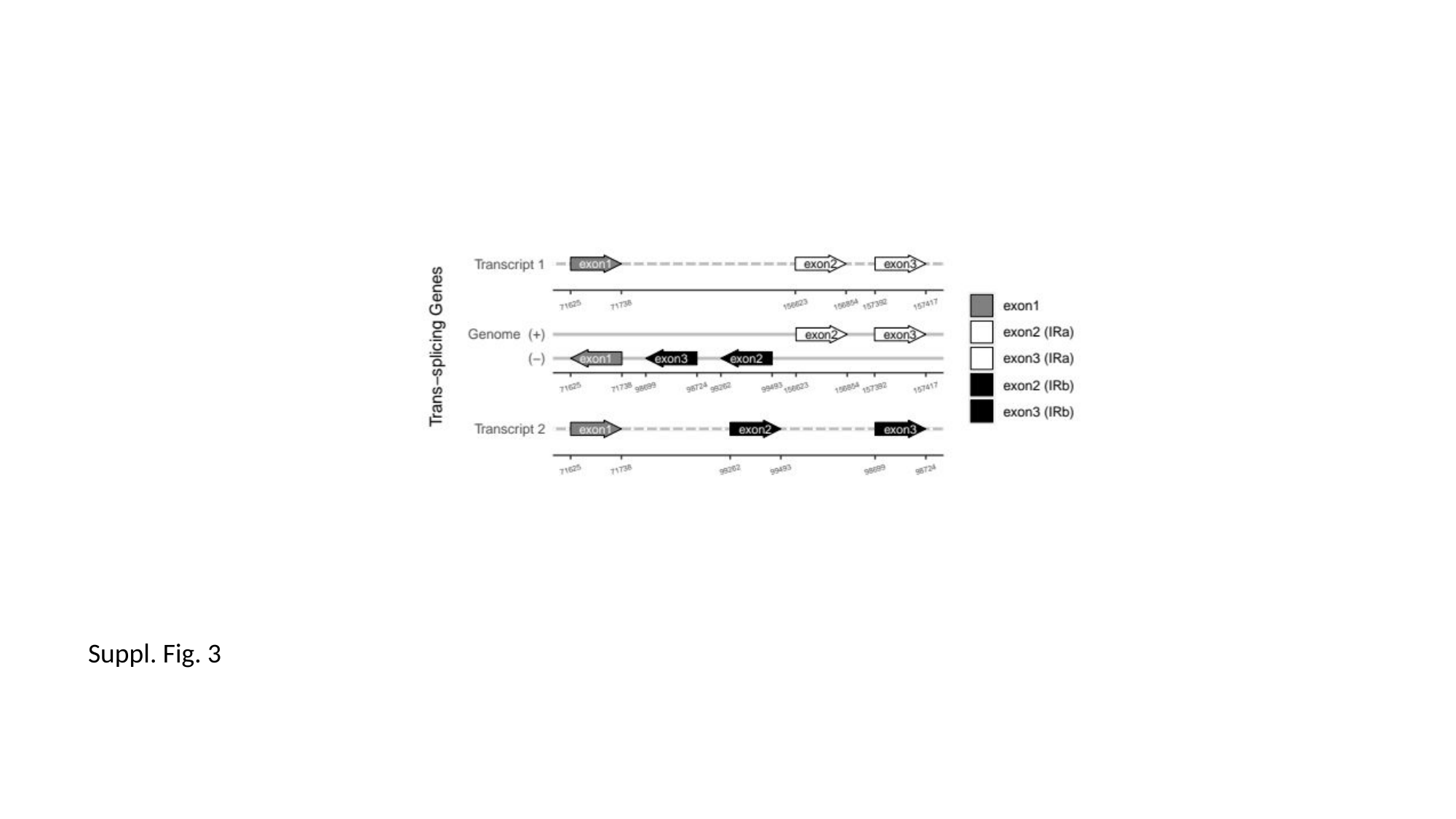

Suppl. Fig. 3

Supplement: Supplemental Material [file TMDN_A_2179356_SM2436.pptx]
